# Measurement properties of the Danish version of the Awareness and Beliefs about Cancer (ABC) measure

**DOI:** 10.1186/s12874-017-0352-2

**Published:** 2017-04-26

**Authors:** Line Hvidberg, Anette Fischer Pedersen, Christian Nielsen Wulff, Anders Helles Carlsen, Peter Vedsted

**Affiliations:** 1 Research Centre for Cancer Diagnosis in Primary Care (CaP), Research Unit for General Practice, Department of Public Health, Aarhus University, Bartholins Allé 2, 8000 Aarhus C, Denmark; 2 Section for General Medical Practice, Department of Public Health, Aarhus University, Bartholins Allé 2, 8000 Aarhus C, Denmark; 3 Department of Oncology, Aarhus University Hospital, Noerrebrogade 44, 8000 Aarhus C, Denmark

**Keywords:** Cancer, Awareness, Beliefs, Data quality, Validity, Reliability, Factor analysis, Known group comparison

## Abstract

**Background:**

The International Cancer Benchmarking Partnership aims to study international differences in cancer survival and the possible causes. Participating countries are Australia, Canada, Norway, Sweden, Denmark and the UK and a particular focus area is differences in awareness and beliefs about cancer. In this connection, the Awareness and Beliefs about Cancer (ABC) measure has been translated into multiple languages. The aim of this study is to appraise the translation process and measurement properties of the Danish version of the ABC measure.

**Methods:**

The translation process included forward and backward translations and a pilot-test. Data quality was assessed using survey data from 3000 Danish respondents and content validity indexes were calculated based on judgments from ten academic researchers. Construct validity was determined by a confirmative factor analysis (CFA) and exploratory factor analyses (EFA) using survey data and a known group comparison analysis including 56 persons. Test-retest reliability was assessed based on responses from 123 person whom completed the interview twice with an interval of 2–3 weeks.

**Results:**

The translation process resulted in a Danish ABC measure conceptually equivalent to the English ABC measure. Data quality was acceptable in relation to non-response to individual items which was maximum 0.3%, but the percentage of respondents answering ‘don’t know’ was above 3% for 16 out of 48 items. Content validity indexes showed that items adequately reflected and represented the constructs to be measured (item content validity indexes: 0.9–1.0; construct content validity indexes: 0.8–1.0). The hypothesised factor structure could not be replicated by a CFA, but EFA on each individual subscale showed that six out of seven subscales were unidimensional. The ABC measure discriminated well between non-medical academics and medical academics, but had some difficulties in discriminating between educational groups. Test–retest reliability was moderate to substantial for most items.

**Conclusions:**

The Danish ABC measure is a useful measurement that is accepted and understood by the target group and with accepted measurement criteria for content validity and test-retest reliability. Future studies may further explore the factorial structure of the ABC measure and should focus on improving the response categories.

**Electronic supplementary material:**

The online version of this article (doi:10.1186/s12874-017-0352-2) contains supplementary material, which is available to authorized users.

## Background

During the last two decades, several studies have shown that the United Kingom (UK) and Denmark have higher cancer incidence and lower survival than other high-income countries [1–3]. In response, the International Cancer Benchmarking Partnership (ICBP) was launched in 2009 to study variations related to cancer survival between Australia, Canada, Norway, Sweden, Denmark and the UK [4]. A particular focus area is differences in awareness and beliefs about cancer, as a possible contributor to the observed differences in cancer survival. In this connection, the Awareness and Beliefs about Cancer (ABC) measure was developed [4], which is an extension of the Cancer Awareness Measure (CAM) [5]. The new items have been adapted from population-based surveys and from studies on cancer beliefs, screening uptake and healthcare seeking [6–8].

The target population for the ABC measure is the adult general population in the participating ICBP countries and the ABC measure was developed to be administered by telephone interview. The measurement aim of the ABC measure is discriminative, thus to differentiate between countries and socio-economic groups in terms of awareness and beliefs about cancer.

The English ABC measure has shown to have acceptable content validity and test-retest reliability, and much effort have been made to obtain conceptual and cultural equivalent Danish, Swedish, Norwegian and Canadian French translations of the ABC measure [4]. However, as noted by Simon et al. [4], measurement properties of the ABC measure need to be established in each country where it is used. Thus, the aims of this study are:

1. To describe the translation process from the English to the Danish ABC measure.
2. To evaluate the data quality of the Danish ABC measure.
3. To evaluate content and construct validity and test-retest reliability of the Danish ABC measure.

## Methods

**Fig. 1:**
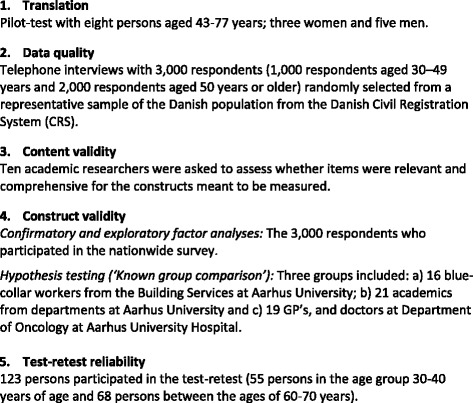
Overview of participants in the study

The translation process and appraisal of the measurement properties can be divided into five steps: (1) translation, (2) data quality, (3) content validity, (4) construct validity and (5) test-retest reliability. An overview of participants in each step is shown in Fig. 1.

### Translation

To achieve a Danish version conceptually equivalent to the English ABC measure the translation was conducted in agreement with the guidelines for translation procedures suggested by de Vet et al. [9]. It involved forward and backward translations with consensus meetings and a pilot-test.

#### Forward translation

Forward translations were performed independently by two native speakers of Danish; one professional translator and one with familiarity with the cancer research area. Based on a consensus meeting with the translators and experts in the cancer area on awareness and beliefs, one reconciled forward version was formed.

#### Backward translation

The reconciled version was back-translated by two native English-speaking persons who were fluent in Danish. The translations were performed independently of each other. Following the back-translation, both translators were provided with the original English version and on a consensus meeting discrepancies between the translations and potential cross-cultural issues were discussed to obtain conceptually equivalent versions of the original English and the Danish ABC measure. The results of the translation were dicussed with the English ICBP group before a pre-final Danish ABC version was made.

#### Pilot-test

The pilot-test took place at the Department of Orthopedic Surgery (foot/ankle and spine sectors) at Aarhus University Hospital. We assumed that persons hospitalised here could use the requested time for an interview and that we would be able to obtain interviews with persons from diverse groups in terms of age, gender, marital status, education and occupation. Hence, three women and five men aged 43–77 years with diverse socio-economic characteristics participated in the pilot-test by means of face-to-face interviews.

First, the interviewer read the related introduction and item to the participants and subsequently methods such as think-aloud and probing were used e.g. “How did you reach the number of days in relation to how long it would take you to go to the doctor?” and “Can you tell me in your own words what you understand by any breast changes?”. Also, participants’ elaborations on difficulties in answering and anything else that shed light on the acceptability and understanding of the measure was noted by the interviewer. The length of the interviews was approximately 1 h.

### Data quality

Data quality reflects respondents’ understanding and acceptance of the items [10]. Data quality was assessed using the Danish data from the ICBP survey. The data collection is described briefly here and for further details see Hvidberg et al. [11]. Between 31 May and 4 July 2011, 3000 Danish residents aged 30 years or older answered the 20-min computer-assisted ABC telephone interview. The respondents’ mean age was 56 years (range 30–99 years) and the majority were women (55%), married/cohabiting (77%) and in the labour force (63%). This information was obtained through individual linkage to Statistics Denmark [12].

For each item the percentage of respondents answering ‘don’t know’ or not answering at all was examined. Less than 3% was considered acceptable [9]. Further, the distribution of responses for each item was examined. Items for which more than 95% of all respondents answered in the same response category was considered to have poor discriminative ability [13].

### Content validity

Content validity assessment examines the degree to which the items adequately reflect and comprehensively represent the construct to be measured [9]. The content validity assessment was based on judgments from ten academic researchers, which is believed to be a sufficient number to provide a sufficient level of control for chance agreement [14]. The researchers had a background in Psychology, Public Health Science or Medicine and had expertise in creating and validating measurements and in cancer and public health research. The content validity assessment was carried out in November 2014.

The content validity index (CVI) was used [14, 15] consisting of the ‘item CVI’ (I-CVI) and ‘construct CVI’ (C—CVI). To calculate the I-CVI, the raters were asked to rate the relevance of each item on a 4-point scale (1: not relevant, 2: somewhat relevant, 3: quite relevant, 4: highly relevant) [15]. Raters were asked for additional comments if they scored an item 1 or 2. For each item, the I-CVI was computed as the number of raters giving a rating of 3 or 4, divided by the total number of raters. Thus, an item rated as ‘quite relevant’ or ‘highly relevant’ by eight out of ten raters would have an I-CVI of 0.80 and an I-CVI of ≥0.80 was considered acceptable for content validity to be established in this study [14].

For calculating C-CVI, the raters were asked to rate the degree to which each construct was covered by the given items on a 4-point scale (1: to a very low degree, 2: to a low degree, 3: to some degree, 4: to a high degree). The same method and criterion as for I-CVI was used for the C-CVI.

### Construct validity

The core ABC measure includes the following five subscales: anticipated patient interval for healthcare seeking (4 items); awareness of cancer symptoms (1 recall item and 11 recognition items) [16]; anticipated barriers for healthcare seeking (4 items); beliefs about cancer (6 items) and awareness of 5-year survival from cancer (4 items). Denmark and some of the other countries from the ICBP included three additional subscales, i.e. beliefs about breast cancer screening (3 items for women only); beliefs about bowel cancer screening (3 items) and awareness of risk factors for cancer (13 recognition items). For this study, construct validity was evaluated by assessing two aspects: structural validity and hypotheses testing [9].

#### Structural validity

Structural validity, i.e. the degree to which the scores on the measure are an adequate reflection of the dimensionality of the construct [9]. The data from the 3000 respondents was used. First, Confirmative Factor Analysis (CFA) was performed as we had a priori hypotheses about which items belonged to which factor. Next, Exploratory Factor Analysis (EFA) was used on the individual subscales to test the extent to which the items in each subscale appeared to represent the same underlying construct.

The subscale ‘awareness of 5-year survival from cancer’ was not included in neither the CFA nor the EFA as these four items are not expected to correlate, as respondents are asked to state how many out of 10 persons are alive after 5 years for four very different types of cancer.

The following three fit indices were applied for CFA and EFA: The root mean square error of approximation (RMSEA; acceptable values < 0.05), the comparative fit index (CFI; acceptable fit > 0.90 and preferable fit > 0.95) and the Tucker-Lewis Index (TLI; acceptable fit > 0.90 and preferable fit > 0.95) [17]. For EFA, oblique rotation was chosen to clarify the data structure and factors were assessed by examine multiple criteria i.e. the Kaiser-Guttman eigenvalues > 1 rule, the scree plot, the factor loading criteria of 0.3 and interpretability of resulting factors. Also crossloadings of 0.3 or higher were assessed [17, 18]. The CFA and EFA were conducted using the WLSMV estimator in Mplus Version 7.4 [19].

#### Hypotheses testing

We tested predefined hypotheses about differences in awareness and beliefs about cancer between different groups regarding educational level or medical proficiency. Three groups were included for this ‘known group comparison’: 1) blue-collar workers at Building Service at Aarhus University; 2) academics at departments at Aarhus University 3) general practitioners (GP’s) and doctors at Department of Oncology at Aarhus University Hospital. All were invited by e-mail and were asked to write back if they agreed to participate. Subsequently, a day and time was arranged where they were called to answer the ABC measure. The data was collected by three unaffiliated trained interviewers between November 2012 and January 2014.

Comparison between groups was made on items where differences were expected based on the literature on awareness and beliefs about cancer [5, 20–24]. Thus, the hypotheses had been formulated a priori based on previous research among different socioeconomic groups in the general population and based on comparisons between cancer experts and non-medical academics [5, 20–24]. Differences in proportions between groups were tested using Fisher’s Exact Test. The statistical significance level was set to 0.05 or less.

The following hypotheses were tested:

Group 1 vs. 2:

I. Awareness of cancer symptoms: Group 2 was expected to be significantly more aware that unexplained bleeding can be a sign of cancer.
II. Anticipated barriers for healthcare seeking: Group 2 was expected to be significantly more likely to concur that being too busy to make time to go to the doctor is a barrier to healthcare seeking.
III. Awareness of risk factors for cancer: Group 2 was expected to be significantly more aware of the risk factor ‘having a close relative with cancer’.
IV. Awareness of risk factors for cancer: Group 2 was expected to be significantly more aware of the risk factor ‘getting sunburnt more than once as a child’.

Group 2 vs. 3:

I. Awareness of cancer symptoms: Group 3 was expected to be significantly more aware that a sore that does not heal can be a sign of cancer.
II. Awareness of 5-year survival from cancer: Group 3 was expected to be significantly more likely to correctly identify the 5-year survival from ovarian cancer.
III. Awareness of risk factors for cancer: Group 3 was expected to be significantly more likely to correctly identify that cancer risk is higher in people aged 70-years than at a younger age.
IV. Awareness of risk factors for cancer: Group 3 was expected to be significantly more likely to correctly identify that ‘infection with human papillomavirus (HPV)’ is a risk factor for cancer.

We used the criterion by Terwee et al. [10] that confirmation of at least 75% of the hypotheses indicates sufficient construct validity.

### Test-retest reliability

The reproducibility, i.e. the degree to which scores are stable over time when the factors underlying the measure have not changed, was assessed with a test-retest [9]. We contacted individuals aged 30–40 years and 60–70 years, who were randomly selected among persons who had not participated in the nationwide survey, but who had been eligible for participation [11]. The ABC measure was completed twice with an interval of 2–3 weeks in the period from March to June 2012. The interval was chosen as an adequate time interval for respondents not to precisely recall their previous responses to the items and for their awareness and beliefs about cancer not to have radically changed between the two occasions. A transition question was included at the end of the retest interview asking whether respondents themselves thought that their awareness and beliefs about cancer had changed since the first interview. If transition had taken place, the respondent was excluded from the test-retest analyses.

A total sample size of 100 persons was determined as reasonable for the test-retest [9]. The test-retest was undertaken by two unaffiliated trained interviewers. The open-ended recall question on symptoms of cancer was not assumed to be stable between test and retest because this question was asked before the 11 closed recognition items on awareness of cancer symptoms.

Test-retest reliability was calculated as the unweighted Cohen’s kappa for nominal items and the quadratic weighted kappa coefficient for ordinal items. Kappa coefficients were interpreted according to Landis and Koch: <0.00 as poor, 0.00–0.20 as slight, 0.21–0.40 as fair, 0.41–0.60 as moderate, 0.61–0.80 as substantial and 0.81–1 as almost perfect agreement [25].

For awareness of cancer symptoms and risk factors for cancer aggregated continuous scores were also computed as papers on the CAM and the ABC measure commonly report a total score of correctly identified symptoms and risk factors [4, 5, 20]. For awareness of cancer symptoms a score of 1 point was given for the answer ‘yes’ and 0 for the answer ‘no’ (possible range of aggregated score: 0–11). For awareness of risk factors for cancer the answers ‘tend to agree’ and ‘strongly agree’ were given 1 point and ‘strongly disagree’ and ‘tend to disagree’ were given 0 points (possible range of aggregated score: 0–13). The intraclass correlation coefficient (ICC) with 95% CI was computed for the total number of symptoms and risk factors recognised using a two-way random effect model measuring absolute agreement (ICC_2,1_ according to Shrout and Fleiss [26]). Guidelines for the interpretation of ICC suggest that a value > 0.70 is acceptable [9]. Subgroup analyses were performed to assess consistency of the kappa coefficients and the ICCs across the two age strata, 30–40 and 60–70 years of age.

## Results

### Translation

#### Final version

The comprehensive translation procedures resulted in a Danish ABC measure that was found to be conceptually equivalent to the English ABC measure. As a consequence of the pilot-test the introduction to the ABC measure was shortened and explanations of some terms were needed (e.g. processed meat). These alterations were incorporated in both the original English version and the Danish version of the ABC measure. Documentation of the translation process is available upon request.

### Data quality

The percentage of respondents answering ‘don’t know’ for each item ranged from 0 to 68.3% and was non-acceptable (above 3%) for 16 out of 48 items (only one item was >10%). The data quality for the 16 items is seen in Table 1 (the data quality for all 48 items can be found in Additional file 1, available online). Non-response to individual items was maximum 0.3%. All items, except for one had acceptable discriminative ability. The item with poor discriminative ability was ‘Change in the appearance of a mole’ as it was recognised as a possible sign of cancer by 97.2% of all respondents (data not shown).

**Table 1:** Data quality: Number of respondents who ‘did not answer’ and who answered ‘don’t know’. Only items with >3% of respondents answering ‘don’t know’ are shown. Total *n* = 3000 for all items

	Did not answer % (*n*)	Don’t know^a^ % (*n*)
Awareness of cancer symptoms		
Response options: yes; no.		
Q10. Persistent unexplained pain	0 (0)	3.4 (101)
Q11. Unexplained bleeding	0 (0)	4.5 (135)
Q14. Persistent difficulty in swallowing	0 (0)	3.4 (101)
Q16. Sore that does not heal	0 (1)	4.5 (134)
Q17. Unexplained night sweats	0 (0)	8.2 (245)
Beliefs about cancer		
Response options: strongly disagree; tend to disagree; tend to agree; strongly agree.		
Q29. Most cancer treatment is worse than the cancer itself	0.3 (8)	9.9 (296)
Awareness of 5-year survival from cancer		
Response options: 0; 1; 2; 3; 4; 5; 6; 7; 8; 9; 10.		
Q34. Out of 10 people diagnosed with bowel cancer, how many do you think would be alive 5 years later?	0.1 (3)	5.0 (151)
Q36. Out of 10 people diagnosed with ovarian cancer, how many do you think would be alive 5 years later?	0.2 (5)	6.3 (189)
Q37. Out of 10 people diagnosed with lung cancer, how many do you think would be alive 5 years later?	0.2 (5)	3.1 (93)
Awareness of risk factors for cancer		
Response options: strongly disagree; tend to disagree; tend to agree; strongly agree		
QN3. Drinking more than 1 unit of alcohol a day	0 (0)	3.1 (94)
QN5. Eating red or processed meat once a day or more	0 (1)	7.5 (224)
QN6. Being obese	0 (1)	4.1 (124)
QN8. Being over 70 years old	0 (1)	3.2 (96)
QN9. Having a close relative with cancer	0.1 (2)	3.1 (94)
QN10. Infection with HPV, Human Papillomavirus	0 (0)	68.3 (2050)
QN13. Exposure to ionising radiation from, for example, radioactive materials, x-rays or radon	0.1 (2)	3.8 (114)

^a^Don’t know was not provided as a response option in any items, but was noted by the interviewer when respondents answered ‘don’t know’ unprovokedly

### Content validity

The majority of the items received high ratings from the ten raters in terms of being relevant for the construct to be measured. Thus, the I-CVI ranged from 0.9 to 1.0. Also, the comprehensiveness of each construct (C-CVI) was given a high rating, ranging from 0.8 to 1.0. The construct given the lowest C-CVI was ‘anticipated patient interval for healthcare seeking’ (data not shown).

### Construct validity

#### Structural validity

The hypothesised seven factor structure of the ABC measure showed a good fit for the RMSEA indice and a poor fit for the other two indices. The indices for model fit were 0.032, 0.864 and 0.854 for RMSEA, CFI and TLI, respectively. Sub-group analyses were performed for men and women separately, because items about breast cancer were only answered by women. This did not change the fit indices significantly.

EFAs on each individual subscale revealed that six out of seven subscales were unidimensional based on the evaluation of eigenvalues, the scree plot, factor loadings and the interpretability of the factors. The subscale that was not unidimensional was ‘Beliefs about cancer’, which showed a two-factor structure as two of its items (‘Q29. Most cancer treatment is worse than the cancer itself’ and ‘Q30. Not want to know if I have cancer’) loaded onto a second factor. Item ‘Q33. A diagnosis of cancer is a death sentence’ also cross-loaded onto this second factor. Considering this and the interpretability of the factor structure it is advocated that the ‘beliefs about cancer’ subscale are split into ‘positive beliefs about cancer’ (item Q28, Q31, Q32) and ‘negative beliefs about cancer’ (item Q29, Q30, Q33). Table 2 presents the results of the EFAs with factor loadings and crossloadings of 0.3 or higher for each item and the goodness of fit indices for each factor.

**Table 2:** Factor loadings of the items in the ABC measure based on EFA for each individual subscale (the loadings in bold are advocated as the final structure of the EFA)

Subscale and items	Factor loadings							
	Factor 1	Factor 2	Factor 3	Factor 4	Factor 5	Factor 6	Factor 7	Factor 8
Anticipated patient interval for healthcare seeking								
Q5. A persistent cough	**0.641**							
Q6. Rectal bleeding	**0.581**							
Q7. Any breast changes^a^	**0.609**							
Q8. Abdominal bloating	**0.646**							
Awareness of cancer symptoms								
Q9. Unexplained lump or swelling		**0.575**						
Q10. Persistent unexplained pain		**0.610**						
Q11. Unexplained bleeding		**0.594**						
Q12. Persistent cough or hoarseness		**0.713**						
Q13. Change in bowel or bladder habits		**0.637**						
Q14. Persistent difficulty in swallowing		**0.696**						
Q15. Change in the appearance of a mole		**0.605**						
Q16. Sore that does not heal		**0.572**						
Q17. Unexplained night sweats		**0.515**						
Q18. Unexplained weight loss		**0.713**						
Q19. Unexplained tiredness		**0.702**						
Anticipated barriers for healthcare seeking								
Q24. I would be too embarrassed			**0.874**					
Q25. I would be worried about what the doctor might find			**0.630**					
Q26. I would be worried about wasting the doctor’s time			**0.379**					
Q27. I am too busy to make time to go to the doctor			**0.402**					
Beliefs about cancer								
Q28. People with cancer can expect to continue with normal activities				**0.605**				
Q29. Most cancer treatment is worse than the cancer itself					**0.410**			
Q30. Not want to know if I have cancer					**0.533**			
Q31. Cancer can often be cured				**0.708**				
Q32. Going to the doctor as quickly as possible could increase the chances of surviving				**0.431**				
Q33. A diagnosis of cancer is a death sentence				0.451	**(0.312)**			
Beliefs about breast cancer screening^a^								
QM3. So worried about what might be found at breast cancer screening, that I would prefer not to do it						**0.628**		
QM4. Breast cancer screening is only necessary if I have symptoms						**0.911**		
QM5. Breast cancer screening could reduce my chances of dying from breast cancer						**0.465**		
Beliefs about bowel cancer screening								
QM6. So worried about what might be found at bowel cancer screening, that I would prefer not to do it							**0.643**	
QM7. Bowel cancer screening is only necessary if I have symptoms							**0.570**	
QM8. Bowel cancer screening could reduce my chances of dying from bowel cancer							**0.400**	
Awareness of risk factors for cancer								
QN1. Smoking								**0.670**
QN2. Exposure to passive smoking								**0.574**
QN3. Drinking more than 1 unit of alcohol a day								**0.521**
QN4. Eating less than 5 portions of fruit and vegetables a day								**0.535**
QN5. Eating red or processed meat once a day or more								**0.483**
QN6. Being obese								**0.537**
QN7. Getting sunburnt more than once as a child								**0.442**
QN8. Being over 70 years old								**0.428**
QN9. Having a close relative with cancer								**0.349**
QN10. Infection with HPV, Human Papillomavirus								**0.468**
QN11. Not doing much physical activity								**0.583**
QN12. Using a solarium								**0.439**
QN13. Exposure to ionising radiation from, for example, radioactive materials, x-rays or radon								**0.406**

^a^Only answered by women

Factor 1: RMSEA: 0.154; CFI: 0.937; TLI: 0.812

Factor 2: RMSEA: 0.025; CFI: 0.983; TLI: 0.978

Factor 3: RMSEA: 0.028; CFI: 0.991; TLI: 0.974

Factor 4/5: RMSEA: 0.012; CFI: 0.999; TLI: 0.995 (two-factor model)

Factor 6: RMSEA: 0.000; CFI: 1.000; TLI: 1.000

Factor 7: RMSEA: 0.000; CFI: 1.000; TLI: 1.000

Factor 8: RMSEA: 0.075; CFI: 0.857; TLI: 0.829

#### Hypotheses testing

In total, 59 persons participated. Three persons which should represent the blue-collar group had first or second stage of tertiary education and where therefore excluded. Ultimately, 56 persons were included in the analysis: 16 blue-collar workers from the Building Service (group 1), 21 academics from departments at Aarhus University (group 2) and 19 GP’s and oncologist (group 3). The mean ages of the three groups were 54, 46 and 50 years for group 1, 2 and 3, respectively. A majority of group 3 were men (74%) compared to 63 and 52% in group 1 and 2, respectively. Group 3 had considerably fewer close relatives with cancer (68%) than group 1 (94%) and group 2 (91%).

Table 3 displays the results of the hypothesis testing. When the two different educational groups (group 1 vs. group 2) were compared three out of four of the hypotheses tested differed in the expected direction, but only one out of four differed statistically significantly. When non-medical and medical academics (group 2 vs. group 3) were compared all of the hypotheses tested differed in the expected direction and three out of four (75%) of the hypotheses differed statistically significantly.

**Table 3:** Hypothesis testing by known group comparison

	Group 1 Blue-collar workers (*n* = 16)		Group 2 Non-medical academics (*n* = 21)		Group 3 GP’s and oncologists (*n* = 19)		*p*-value^a^
	%	(*n*)	%	(*n*)	%	(*n*)	
Hypothesis: Group 2 > group 1							
Awareness of unexplained bleeding	81.3	(13)	61.9	(13)	-	-	0.285
Hypothesis: Group 2 > group 1							
Being too busy to make time to go to the doctor^b^	12.5	(2)	47.6	(10)	-	-	*0.035*
Hypothesis: Group 2 > group 1							
Awareness of having a close relative with cancer^c^	62.5	(10)	81.0	(17)	-	-	0.274
Hypothesis: Group 2 > group 1							
Awareness of getting sunburnt more than once as a child^c^	56.3	(9)	71.4	(15)			0.489
Hypothesis: Group 3 > group 2							
Awareness of a sore that does not heal	-	-	52.4	(11)	100	(19)	*0.001*
Hypothesis: Group 3 > group 2							
Correctly identifying the 5-year survival from ovarian cancer^d^	-	-	9.5	(2)	57.9	(11)	*0.002*
Hypothesis: Group 3 > group 2							
Correctly identifying that cancer risk is higher in people aged 70-years than at a younger age	-	-	71.4	(15)	94.7	(18)	0.095
Hypothesis: Group 3 > group 2							
Awareness of infection with human papillomavirus (HPV) ^c^	-	-	47.6	(10)	100	(19)	*0.000*

^a^Fischer’s exact test. Statistical significance, italics *p*-value < 0.05

^b^Response options were yes often, yes sometimes and no, which were dichotomised into yes/no

^c^Response options were strongly disagree, tend to disagree, tend to agree and strongly agree, which were dichotomised into disagree/agree

^d^For ovarian cancer an answer of 3 or 4 out of 10 was coded as correct

### Test-retest reliability

**Fig. 2:**
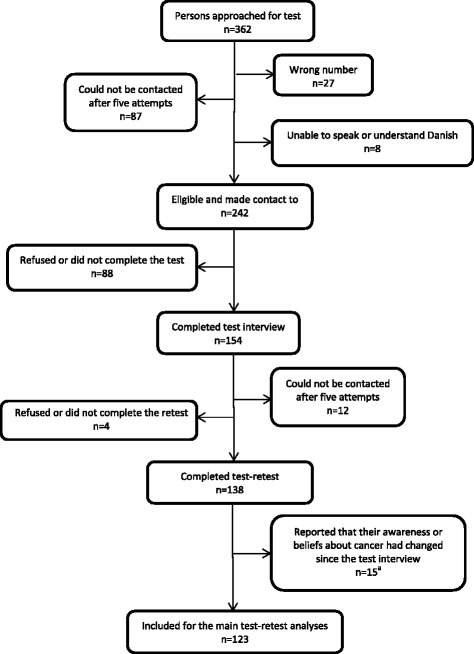
Flowchart of participants in test-retest. ^a^Respondents were asked to what degree their awareness or beliefs about cancer had changed: To a high degree (*n* = 1), to some degree (*n* = 1) and to a minor degree (*n* = 13)

Figure 2 shows the flowchart for test-retest. Of 362 persons approached for participation in the test-retest, 138 (38%) persons answered both the test and the retest. Fifteen persons were excluded as they reported a change in their awareness and/or beliefs about cancer leaving 123 persons (34%) for the analyses.

Results of the test-retest reliability are shown in Table 4. The percentage of agreement between test and retest ranged from 77.6 to 100% with 48 out of 56 items having an agreement of >90%. The kappa coefficient ranged from −0.01 (*change in the appearance of a mole*) to 1 (*bowel cancer screening behavior*) and most of the kappa coefficients were in the range moderate to substantial (0.41–0.80). The ICC for the aggregated scores for awareness of cancer symptoms and risk factors for cancer were 0.80 and 0.75, respectively.

**Table 4:** Test-retest reliability of the ABC measure

	*N*	Agreement (%)	Expected agreement (%)	Kappa	ICC (95% CI)
Anticipated patient interval for healthcare seeking					
Response options: I would go as soon as I noticed; up to 1 week; over 1 up to 2 weeks; over 2 up to 3 weeks; over 3 up to 4 weeks; more than a month; I would go to another healthcare professional; I would not contact my doctor.					
Q5. A persistent cough	119	95.6	87.3	0.65	
Q6. Rectal bleeding	120	98.5	92.7	0.80	
Q7. Any breast changes^b^	75	95.4	92.0	0.43	
Q8. Abdominal bloating	115	94.2	80.4	0.70	
Awareness of cancer symptoms					
Response options: yes and no.					
Q9. Unexplained lump or swelling	122	90.2	87.7	0.20	
Q10. Persistent unexplained pain	116	77.6	62.3	0.41	
Q11. Unexplained bleeding	116	88.8	78.1	0.49	
Q12. Persistent cough or hoarseness	119	84.9	63.9	0.58	
Q13. Change in bowel or bladder habits	118	88.1	68.6	0.62	
Q14. Persistent difficulty in swallowing	117	90.6	67.9	0.71	
Q15. Change in the appearance of a mole	123	98.4	98.4	−0.01	
Q16. Sore that does not heal	107	82.2	65.4	0.49	
Q17. Unexplained night sweats	100	81.0	63.4	0.48	
Q18. Unexplained weight loss	119	95.0	84.6	0.67	
Q19. Unexplained tiredness	115	86.1	65.0	0.60	
The total score of cancer symptom awareness	123	-	-	-	0.80 (0.72–0.86)
Anticipated barriers for healthcare seeking					
Response options: yes, often; yes, sometimes; no.					
Q24. I would be too embarrassed	123	98.0	92.6	0.72	
Q25. I would be worried about wasting the doctor’s time	123	98.0	91.0	0.77	
Q26. I would be worried about what the doctor might find	123	95.9	88.0	0.66	
Q27. I am too busy to make time to go to the doctor	123	96.3	87.7	0.70	
Beliefs about cancer					
Response options: strongly disagree; tend to disagree; tend to agree; strongly agree.					
Q28. People with cancer can expect to continue with normal activities	119	93.2	88.2	0.42	
Q29. Most cancer treatment is worse than the cancer itself	103	93.3	81.0	0.65	
Q30. Not want to know if I have cancer	122	96.7	94.3	0.43	
Q31. Cancer can often be cured	123	94.2	90.9	0.37	
Q32. Going to the doctor as quickly as possible could increase the chances of surviving	122	95.7	93.2	0.37	
Q33. A diagnosis of cancer is a death sentence	122	91.3	84.5	0.44	
Awareness of 5-year survival from cancer					
Response options: 0; 1; 2; 3; 4; 5; 6; 7; 8; 9; 10.					
Q34. Out of 10 people diagnosed with bowel cancer, how many do you think would be alive 5 years later?	112	96.2	93.5	0.42	
Q35. Out of 10 people diagnosed with breast cancer, how many do you think would be alive 5 years later?	120	96.7	91.7	0.60	
Q36. Out of 10 people diagnosed with ovarian cancer, how many do you think would be alive 5 years later?	109	97.5	90.2	0.74	
Q37. Out of 10 people diagnosed with lung cancer, how many do you think would be alive 5 years later?	120	97.8	92.7	0.70	
Breast cancer screening behaviour^b^					
Response options: yes; no.					
QM1. Breast cancer screening behavior	38	97.4	75.2	0.89	
Bowel cancer screening behaviour^c^					
Response options: yes; no.					
QM2. Bowel cancer screening behavior	68	100	83.9	1	
Beliefs about breast cancer screening^a^					
Response options: strongly disagree; tend to disagree; tend to agree; strongly agree.					
QM3. So worried about what might be found at breast cancer screening, that I would prefer not to do it	75	98.4	95.2	0.66	
QM4. Breast cancer screening is only necessary if I have symptoms	75	92.4	78.0	0.66	
QM5. Breast cancer screening could reduce my chances of dying from breast cancer	74	96.7	87.9	0.73	
Beliefs about bowel cancer screening					
Response options: strongly disagree; tend to disagree; tend to agree; strongly agree.					
QM6. So worried about what might be found at bowel cancer screening, that I would prefer not to do it	121	97.0	95.1	0.38	
QM7. Bowel cancer screening is only necessary if I have symptoms	116	87.9	70.8	0.59	
QM8. Bowel cancer screening could reduce my chances of dying from bowel cancer	115	92.8	88.6	0.36	
Awareness of growing risk of cancer with age					
Response options: 30 year olds; 50 year olds; 70 year olds; people of any age are equally likely to be diagnosed with cancer.					
Q38. Growing risk of cancer with age	123	96.5	83.5	0.79	
Awareness of risk factors for cancer					
Response options: strongly disagree; tend to disagree; tend to agree; strongly agree.					
QN1. Smoking	123	98.0	94.8	0.62	
QN2. Exposure to passive smoking	122	97.5	89.5	0.76	
QN3. Drinking more than 1 unit of alcohol a day	118	90.9	81.6	0.50	
QN4. Eating less than 5 portions of fruit and vegetables a day	117	90.1	80.1	0.55	
QN5. Eating red or processed meat once a day or more	109	91.0	80.5	0.54	
QN6. Being obese	117	93.1	81.1	0.63	
QN7. Getting sunburnt more than once as a child	120	92.5	79.0	0.64	
QN8. Being over 70 years old	121	90.4	77.3	0.57	
QN9. Having a close relative with cancer	119	92.1	82.0	0.56	
QN10. Infection with HPV, Human Papillomavirus	37	95.2	87.3	0.62	
QN11. Not doing much physical activity	120	93.8	83.7	0.62	
QN12. Using a solarium	122	97.5	94.3	0.57	
QN13. Exposure to ionising radiation from, for example, radioactive materials, x-rays or radon	119	95.7	91.6	0.49	
The total score of cancer symptom awareness	121	-	-	-	0.75 (0.67–0.82)
Self or someone close with cancer					
Response options: yes, respondent; yes, someone close; yes, both self and someone close; yes, but would prefer not to say who; no.					
Q3. Self or someone close with cancer	123	93.5	58.4	0.84	
Self-rated health					
Response options: very good; good; fair; poor; very poor.					
Q20. Self-rated health	123	97.8	92.2	0.71	
Access to a doctor					
Response options: very difficult; somewhat difficult; somewhat easy; very easy.					
Q21. Access to a doctor	122	97.7	92.4	0.70	
Smoking behavior					
Response options: yes; no.					
Q22. Current smoker	123	100	73.9	1.00	
Q23. Former smoker	104	94.2	50.0	0.88	

^a^Weighted kappa computed for women only

^b^Kappa computed for women ≥ 50 years old

^c^Kappa computed for men and women ≥ 50 years old

The sub-group analyses of the two age strata showed a similar pattern of agreement as that seen for the combined analysis. However due to more homogeneity in the response categories for the oldest age group, the kappa coefficients and the ICCs were generally lower for this group.

## Discussion

The translation and pilot-test procedures resulted in a final version of the Danish ABC measure that was found to be conceptually equivalent to the English ABC measure and that was accepted by the target group. However, evaluation of the data quality showed that the amount of respondents answering ‘don’t know’ was high (above 3%) for 16 out of 48 items. It is an ongoing debate of whether to include a midpoint or neutral response in measures [9]. The ABC measure has no midpoint response and therefore ‘don’t know’ may have been used by respondents when the other response options did not fit their answers or when they did not understand the item or simply did not know the answer.

Only one item, i.e. change in the appearance of a mole, had limited discriminative value given that 97% of respondents concurred this item, however, it was included in the ABC measure because of its importance for face validity [4]. The quantitative content validity assessment showed that items in the ABC measure adequately reflected and represented the constructs to be measured. However, the hypothesized factor structure of the ABC measure could not be replicated by a CFA and the iterative analyses put forward a five-factor structure of the ABC measure. The ABC measure is an extension of the CAM that was developed and validated in 2007–8 [5]. The amendment of subscales on beliefs about cancer and screening for cancer was made in order for the ABC measure to better reflect important determinants for participation in cancer screening and symptom-triggered healthcare seeking. This also means that the ABC measure is not based on a strong theoretical model and the lack of psychometric support for the proposed factor structure may be explained by this fact [17]. It was, however, reassuring that six out of seven subscales were unidimensional in the EFA on each subscale. The EFA on the subscale ‘Beliefs about cancer’suggested two different factors. This finding is not surprising, but in line with a previous study on the Danish ABC measure [27], where we proposed that the positive and negative beliefs about cancer may not be two poles on a unidimensional scale.

The ABC measure discriminated statistically well between the group of non-medical academics and the group of medical academics but not statistically well between the blue-collar workers and the non-medical academics. When comparing blue-collar workers and non-medical academics three of four hypotheses tested differed in the expected direction. However, it was surprising that 80% of the blue-collar workers were aware that unexplained bleeding could be a warning sign for cancer compared to only 60% of the academics. Validation is a continuous process and more research is needed to discover whether the ABC measure is poor at discriminating between various non-medical educational groups concerning awareness of cancer symptoms in a Danish population.

Similar to the generic and several cancer specific versions of the CAM which have been developed and validated [5, 22–24, 28], the Danish version of the ABC measure met accepted psychometric criteria for test-retest reliability. The CAM studies generally found higher values for test-retest and this may among others be due to the fact that all studies except one [28] used the mean of each subscale and Pearson’s correlation, which is not a very stringent parameter to assess test-retest reliability [9]. In our study, respondents were excluded from the test-retest analysis if they indicated a transition of awareness and beliefs about cancer. However, there may have been some unknowingly learning effects, as the aggregated score was higher for both awareness about cancer symptoms and risk factors in the retest. Taking this into account and the fact that even measurement of the most stable factors can be affected by fatigue, motivation and distraction [29] it is sufficient that the majority of the items had an agreement of more than 90%.

The major strength of this study is the systematic way of evaluating the measurement properties of the ABC measure by applying several of the quality criteria for good psychometric properties developed by de Vet et al. [9].

The study also has some limitations which should be noted. First, the data of the 3000 respondents who participated in the nationwide survey was collected in mid-2011. However, data for the content validity assessment, known-group comparison and test-retest were collected afterwards. Thus, results from these analyses have not been used to refine the Danish ABC measure.

Further, in respect to the known group comparison it is a limitation that the three groups differed on other indicators (age, marital status and experience of cancer) than educational level and medical proficiency as we cannot dismiss that this has affected the results. Also, it would have been preferable to have included hypotheses about all items in each construct together with expected magnitudes of the differences for the outcomes and to include discriminative hypotheses between the groups in relation to for example ‘Beliefs about cancer’ and ‘Beliefs about screening for cancer’. However, this was hampered by lack of previous studies providing results on the differences in proportions for outcomes on cancer awareness and lack of pre-existing measures on beliefs about cancer in the literature, respectively [4]. Lastly, data for the known-group comparison was collected in two rounds (November 2012-March 2013 and November 2013-January 2014). The reason was that we had some difficulties in recruiting participants and data collection was put on hold while a Danish cancer awareness campaign was running (March-April 2013) [30]. The two-round data collection was not ideal, but we believe that the interval of about six months between the campaign and the second round of data collection was adequate in order for the results of the known group comparison not to be affected.

## Conclusion and perspectives

The Danish version of the Awareness and Beliefs about Cancer measure appears to be a useful measurement for assessing the Danish population’s awareness and beliefs about cancer. It was accepted and understood by the target group and it met accepted measurement criteria for content validity and test-retest reliability. However, this study also showed some areas in which it can be improved when used in a general population of Danish adults. Hence, future studies may further explore the factorial structure of the ABC measure and should focus on improving the response categories in order to improve the data quality of the measure.

## Additional file

Additional file 1: Response distributions for all 48 items in the Danish ABC measure. This file contains information on the response distribution for each item together with information on non-response and the percentage of respondents answering ‘don’t know’ for each item. (PDF 109 kb)

## Abbreviations

ABC: Awareness and Beliefs about Cancer
CAM: Cancer Awareness Measure
CFA: Confirmative Factor Analysis
CFI: Comparative fit index
CVI: Content validity index
EFA: Exploratory Factor Analysis
GP: General practitioner
ICBP: International Cancer Benchmarking Partnership
ICC: Intraclass correlation coefficient
RMSEA: Root mean square error of approximation
TLI: Tucker-Lewis Index
UK: United Kingdom
