# Supplementary material for: Measurement properties of the Danish version of the Awareness and Beliefs about Cancer (ABC) measure
Source: BMC Med Res Methodol. 2017 Apr 26;17:74. doi: 10.1186/s12874-017-0352-2 (PMC5405495; doi:10.1186/s12874-017-0352-2)
Supplement: Additional file 1: — Response distributions for all 48 items in the Danish ABC measure. This file contains information on the response distribution for each item together with information on non-response and the percentage of respondents answering ‘don’t know’ for each item. (PDF 109 kb) [file 12874_2017_352_MOESM1_ESM.pdf]

Additional file. Response distributions for each items of the eight subscales in the Danish ABC measure.

Anticipated patient interval for healthcare seeking

	N	Did not answer	Don't know	As soon as I noticed	Up to 1 week	Over 1 up to 2 weeks	Over 2 up to 3 weeks	Over 3 up to 4 weeks	More than a month	Go to another healthcare professional	I would not contact my doctor
Q5. A persistent cough	3000	0 (0)	1.6 (47)	6.6 (197)	13.0 (389)	25.0 (750)	16.6 (497)	15.7 (470)	17.9 (538)	0.2 (6)	3.5 (106)
Q6. Rectal bleeding	3000	0 (0)	0.7 (20)	56.6 (1,699)	27.8 (833)	7.9 (237)	2.7 (81)	1.7 (52)	1.9 (57)	0.1 (2)	0.6 (19)
Q7. Any breast changes	1659	0 (0)	0.6 (10)	66.7 (1,107)	19.4 (321)	6.3 (105)	2.2 (37)	1.9 (32)	2.3 (38)	0.2 (3)	0.4 (6)
Q8. Abdominal bloating	3000	0.1 (3)	2.3 (70)	10.0 (300)	21.2 (636)	20.9 (627)	11.7 (350)	10.3 (308)	14.1 (422)	0.2 (6)	9.3 (278)

Awareness of cancer symptoms

	N	Did not answer	Don't know	Yes	No
Q9. Unexplained lump or swelling	3000	0 (0)	0.8 (25)	94.3 (2,829)	4.9 (146)
Q10. Persistent unexplained pain	3000	0 (0)	3.4 (101)	75.3 (2,258)	21.4 (641)
Q11. Unexplained bleeding	3000	0 (0)	4.5 (135)	79.5 (2,385)	16.0 (480)
Q12. Persistent cough or hoarseness	3000	0.1 (4)	1.6 (49)	76.3 (2,290)	21.9 (657)
Q13. Change in bowel or bladder habits	3000	0 (1)	2.5 (74)	80.2 (2,405)	17.3 (520)
Q14. Persistent difficulty in swallowing	3000	0 (0)	3.4 (101)	81.2 (2,437)	15.4 (462)
Q15. Change in the appearance of a mole	3000	0.1 (2)	0.8 (24)	97.2 (2,916)	1.9 (58)
Q16. Sore that does not heal	3000	0 (1)	4.5 (134)	67.8 (2,034)	27.7 (831)
Q17. Unexplained night sweats	3000	0 (0)	8.2 (245)	15.6 (467)	76.3 (2,288)
Q18. Unexplained weight loss	3000	0 (0)	1.2 (35)	91.9 (2,756)	7.0 (209)
Q19. Unexplained tiredness	3000	0 (0)	1.8	80.1 (2,403)	18.1 (543)

Anticipated barriers for healthcare seeking

	N	Did not answer	Don't know	Yes, often	Yes, sometimes	No
Q24. I would be too embarrassed	3000	0 (1)	0 (1)	1.2 (36)	5.4 (162)	93.3 (2,800)
Q25. I would be worried about wasting the doctor's time	3000	0 (0)	0.2 (5)	3.2 (97)	11.5 (346)	85.1 (2,552)
Q26. I would be worried about what the doctor might find	3000	0 (0)	0.5 (14)	5.0 (150)	21.6 (647)	73.0 (2,189)
Q27. I am too busy to make time to go to the doctor	3000	0 (1)	0.1 (2)	6.4 (192)	18.0 (539)	75.5 (2,266)

Beliefs about cancer

	N	Did not answer	Don't know	Strongly disagree	Tend to disagree	Tend to agree	Strongly agree
Q28. People with cancer can expect to continue with normal activities	3000	0 (1)	3.0 (91)	3.0 (89)	10.7 (321)	47.5 (1,424)	25.8 (1,074)
Q29. Most cancer treatment is	3000	0.3 (8)	9.9 (296)	10.7 (321)	19.6 (588)	36.2 (1,086)	23.4 (701)

worse than the cancer itself														
Q30. Not want to know if I have cancer	3000	0.1 (4)	2.1 (63)	77.3 (2,318)	10.5 (314)	4.3 (128)	5.8 (173)							
Q31. Cancer can often be cured	3000	0.1 (4)	1.6 (48)	3.1 (92)	7.6 (228)	46.0 (1,379)	41.6 (1,249)							
Q32. Going to the doctor as quickly as possible could increase the chances of surviving	3000	0 (0)	0.3 (10)	1.3 (39)	1.1 (34)	8.9 (266)	88.4 (2,651)							
Q33. A diagnosis of cancer is a death sentence	3000	0.1 (4)	1.9 (57)	33.4 (1,003)	36.1 (1,084)	22.7 (680)	5.7 (172)							
**Awareness of 5-year survival from cancer**														
	**N**	**Did not answer**	**Don't know**	**0**	**1**	**2**	**3**	**4**	**5**	**6**	**7**	**8**	**9**	**10**
Q34. Out of 10 people diagnosed with bowel cancer, how many do you think would be alive 5 years later?	3000	0.1 (3)	5.0 (151)	1.4 (43)	1.7 (51)	6.4 (192)	8.2 (245)	7.9 (238)	29.8 (895)	10.1 (303)	11.9 (358)	12.7 (381)	3.1 (93)	1.6 (47)
Q35. Out of 10 people diagnosed with breast cancer, how many do you think would be alive 5 years later?	3000	0.1 (3)	2.8 (85)	0.3 (9)	0.3 (10)	0.9 (26)	1.9 (57)	2.2 (67)	14.2 (426)	7.5 (226)	17.7 (530)	31.9 (956)	15.6 (467)	4.6 (138)
Q36. Out of 10 people diagnosed with ovarian cancer, how many do you think would be alive 5 years later?	3000	0.2 (5)	6.3 (189)	0.5 (15)	1.0 (30)	2.7 (82)	4.1 (124)	4.5 (135)	22.3 (669)	7.7 (230)	13.6 (409)	20.6 (617)	10.6 (317)	5.9 (178)
Q37. Out of 10 people diagnosed with lung cancer, how many do you think would be alive 5 years later?	3000	0.2 (5)	3.1 (93)	3.0 (91)	5.8 (173)	12.5 (376)	13.9 (417)	12.1 (363)	31.3 (940)	6.7 (200)	5.3 (158)	4.9 (146)	0.9 (28)	0.3 (10)
**Beliefs about breast cancer screening**														
	**N**	**Did not answer**	**Don't know**	**Strongly disagree**	**Tend to disagree**	**Tend to agree**	**Strongly agree**							
QM3. So worried about what might be found at breast cancer screening, that I would prefer not to do it.	1659	0.3 (5)	0.9 (15)	81.5 (1,352)	9.5 (157)	4.1 (68)	3.7 (62)							
QM4. Breast cancer screening is only necessary if I have symptoms	1659	0.2 (4)	1.2 (19)	67.6 (1,121)	14.6 (242)	8.0 (133)	8.4 (140)							
QM5. Breast cancer screening could reduce my chances of dying from breast cancer	1659	0 (0)	1.4 (23)	4.5 (74)	4.3 (71)	18.9 (314)	71.0 (1,177)							
**Beliefs about bowel cancer screening**														
	**N**	**Did not answer**	**Don't know**	**Strongly disagree**	**Tend to disagree**	**Tend to agree**	**Strongly agree**							
QM6. So worried about what	3000	0.2 (7)	1.4 (43)	75.2 (2,257)	12.9 (387)	5.9 (176)	4.3 (130)							

	N	Did not answer	Don't know	Strongly disagree	Tend to disagree	Tend to agree	Strongly agree
might be found at bowel cancer screening, that I would prefer not to do it							
QM7. Bowel cancer screening is only necessary if I have symptoms	3000	0.3 (9)	3.0 (89)	34.6 (1,038)	20.3 (608)	21.8 (654)	20.1 (602)
QM8. Bowel cancer screening could reduce my chances of dying from bowel cancer	3000	0.3 (8)	2.8 (84)	4.3 (129)	3.8 (113)	24.0 (721)	64.8 (1,945)
**Awareness of risk factors for cancer**							
	**N**	**Did not answer**	**Don't know**	**Strongly disagree**	**Tend to disagree**	**Tend to agree**	**Strongly agree**
QN1. Smoking	3000	0.1 (2)	0.3 (10)	1.7 (52)	1.4 (42)	10.3 (310)	86.1 (2,584)
QN2. Exposure to passive smoking	3000	0 (1)	1.1 (34)	4.1 (123)	7.0 (211)	38.3 (1,148)	49.4 (1,483)
QN3. Drinking more than 1 unit of alcohol a day	3000	0 (0)	3.1 (94)	25.3 (758)	28.3 (848)	28.8 (864)	14.5 (436)
QN4. Eating less than 5 portions of fruit and vegetables a day	3000	0 (0)	2.6 (77)	22.4 (672)	34.0 (1,020)	27.9 (837)	13.1 (394)
QN5. Eating red or processed meat once a day or more	3000	0 (1)	7.5 (224)	15.9 (477)	27.0 (811)	31.4 (941)	18.2 (546)
QN6. Being obese	3000	0 (1)	4.1 (124)	9.7 (291)	19.4 (581)	31.5 (945)	35.3 (1,058)
QN7. Getting sunburnt more than once as a child	3000	0 (1)	3.0 (90)	14.5 (436)	17.5 (526)	35.3 (1,058)	29.6 (889)
QN8. Being over 70 years old	3000	0 (1)	3.2 (96)	19.7 (591)	26.5 (794)	30.0 (900)	20.6 (618)
QN9. Having a close relative with cancer	3000	0.1 (2)	3.1 (94)	12.8 (385)	13.8 (414)	41.7 (1,252)	28.4 (853)
QN10. Infection with HPV, Human Papillomavirus	3000	0 (0)	68.3 (2,050)	3.6 (107)	4.5 (134)	10.0 (299)	13.7 (410)
QN11. Not doing much physical activity	3000	0 (0)	3.0 (89)	12.3 (369)	20.2 (605)	34.1 (1,022)	30.5 (915)
QN12. Using a solarium	3000	0 (0)	0.8 (24)	1.9 (56)	1.9 (56)	14.5 (436)	80.9 (2,428)
QN13. Exposure to ionising radiation from, for example, radioactive materials, x-rays or radon	3000	0.1 (2)	3.8 (114)	2.3 (70)	4.0 (121)	18.5 (556)	71.2 (2,137)
